# Supplementary material for: Candidate Phyla Radiation Roizmanbacteria From Hot Springs Have Novel and Unexpectedly Abundant CRISPR-Cas Systems
Source: Front Microbiol. 2019 May 3;10:928. doi: 10.3389/fmicb.2019.00928 (PMC6509639; doi:10.3389/fmicb.2019.00928)
Supplement: Supplementary file 2 [file Data_Sheet_1.pdf]

Supplementary Figures

for

Candidate Phyla Radiation Roizmanbacteria from hot springs have novel and unexpectedly abundant CRISPR-Cas systems

Lin-Xing Chen[1], Basem Al-Shayeb[2], Raphaël Méheust[1,3], Wen-Jun Li[4], Jennifer A Doudna[3,5] and Jillian F Banfield[1,3*]

[1] Department of Earth and Planetary Sciences, University of California, Berkeley, California 94720, USA.
[2] Department of Plant and Microbial Biology, University of California, Berkeley, California 94720, USA.
[3] Innovative Genomics Institute at UC Berkeley, Berkeley, California 94720, USA.
[4] State Key Laboratory of Biocontrol and Guangdong Provincial Key Laboratory of Plant Resources, School of Life Sciences, Sun Yat-Sen University, Guangzhou 510275, PR China
[5] Department of Chemistry, University of California, Berkeley, California 94720, USA.

[*] Corresponding author: Jillian F Banfield - jbanfield@berkeley.edu

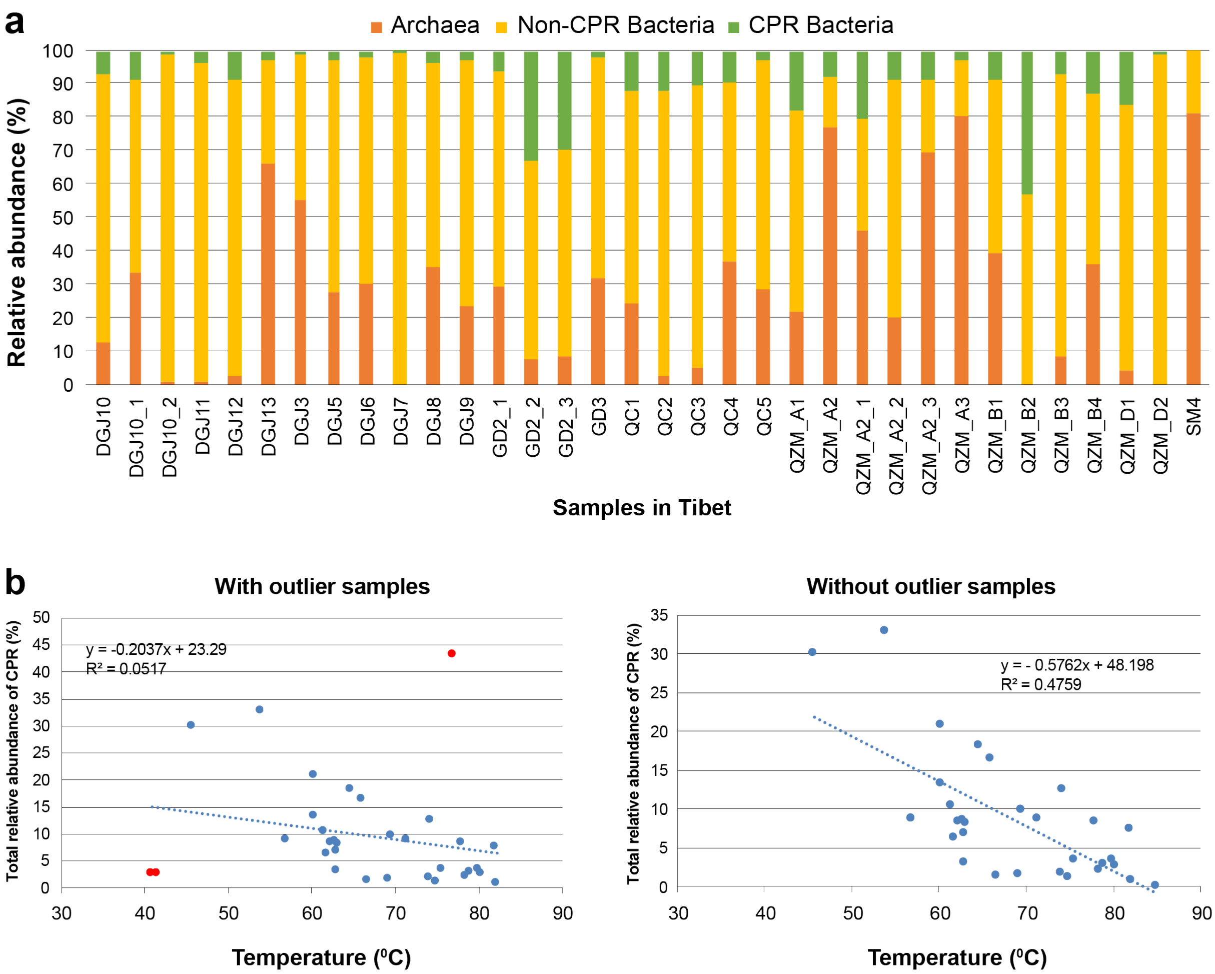

Figure S1 (a) Relative abundance of Archaea, CPR and non-CPR bacteria in the hot spring samples from Tibet. The relative abundance was calculated by (1) identifying all the bacterial and archaeal rpS3 proteins; (2) building a phylogenetic tree with references obtained by BLAST against rpS3 sequences identified in step (1); (3) assigning each rpS3 to a phylum based on the phylogenetic tree; (4) using the coverage of each rpS3, determined by read mapping; (5) calculating the relative abundance of each rpS3 by dividing the total coverage of all rpS3 in a sample; (6) obtaining the total relative abundance of archaea, CPR and non-CPR bacteria. (b) Correlation between temperature and relative abundance of CPR in hot spring samples analyzed in this study. Left panel, all the samples with temperature value available are included. Right panel, three extreme outlier samples (red dots in the left panel) were excluded. Significant correlations could only be detected between temperature and CPR abundance for samples without the three outliers (Pearson's test, P = 0.000013).

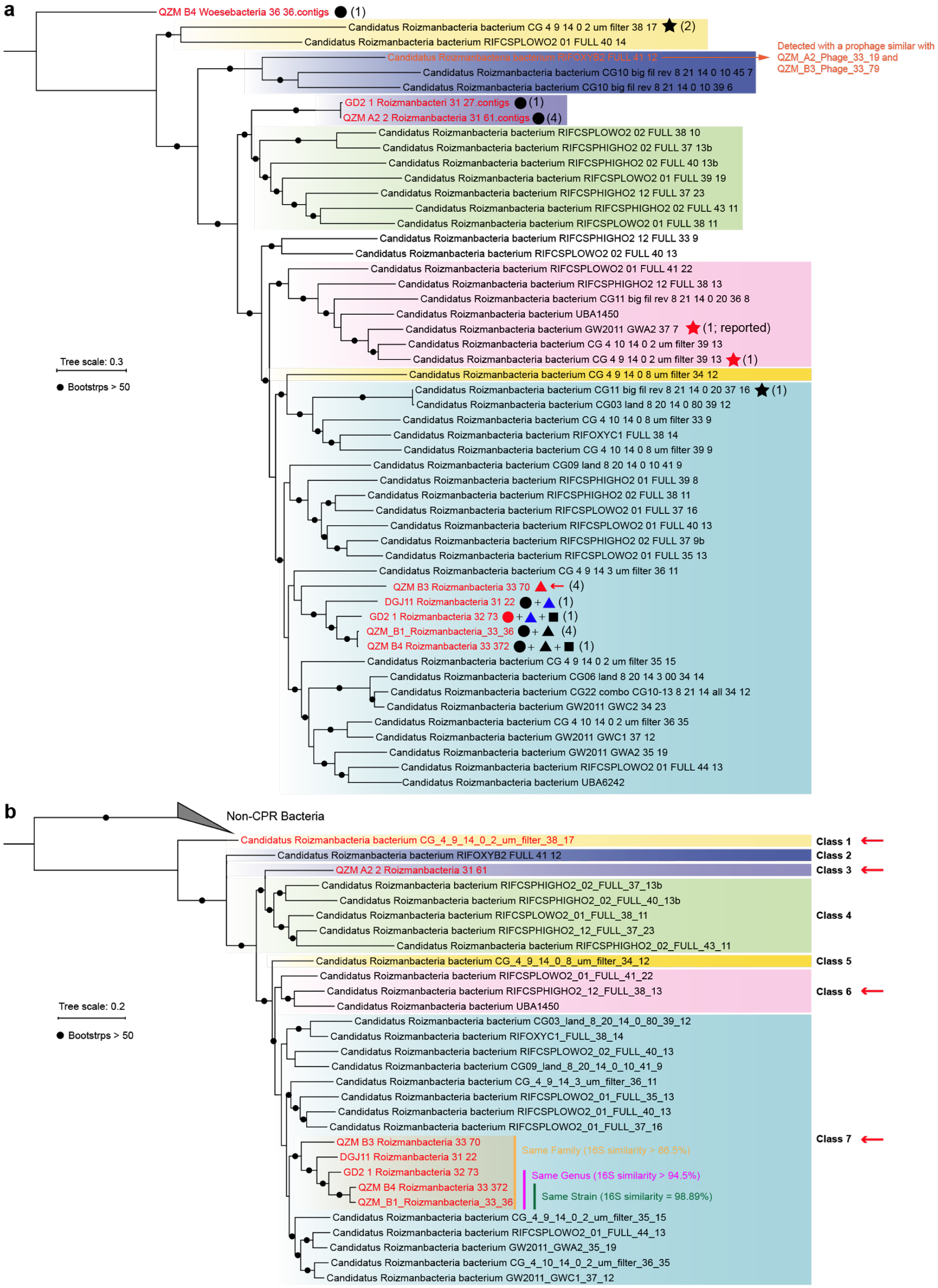

Figure S2 Phylogenetic analyses of Woesebacteria and Roizmanbacteria genomes. (a) A version of Figure 1a with representative genomes with a CRISPR-Cas system shown in red. (b) Phylogenetic tree of genomes in Figure 1a, based on their 16S rRNA gene sequences. Genomes were clustered at different taxonomic levels based on their 16S rRNA gene sequence similarity according to thresholds suggested previously (Yarza et al., 2014).

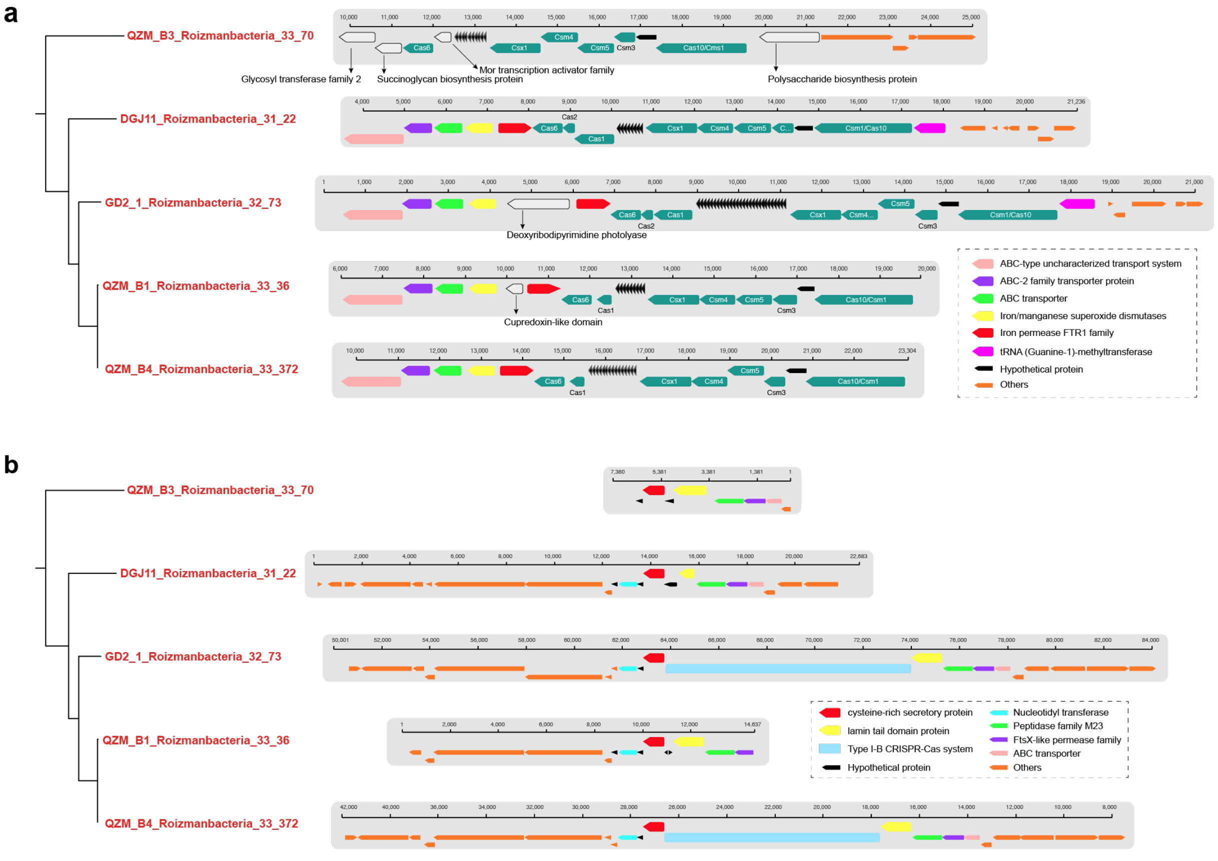

Figure S3 Locations of the CRISPR-Cas (a) Type I-B and (b) III-A systems detected in the Roizmanbacteria genomes. For a given CRISPR-Cas system, if it was detected in a genome, the neighbour genes flanking the systems were predicted and annotated, and compared with those from the same system in other genomes. Homologous genomes are shown in the same colors. The phylogenetic relationships of the genomes were obtained from the 16 rp tree as shown in Figure 1a of the main text.

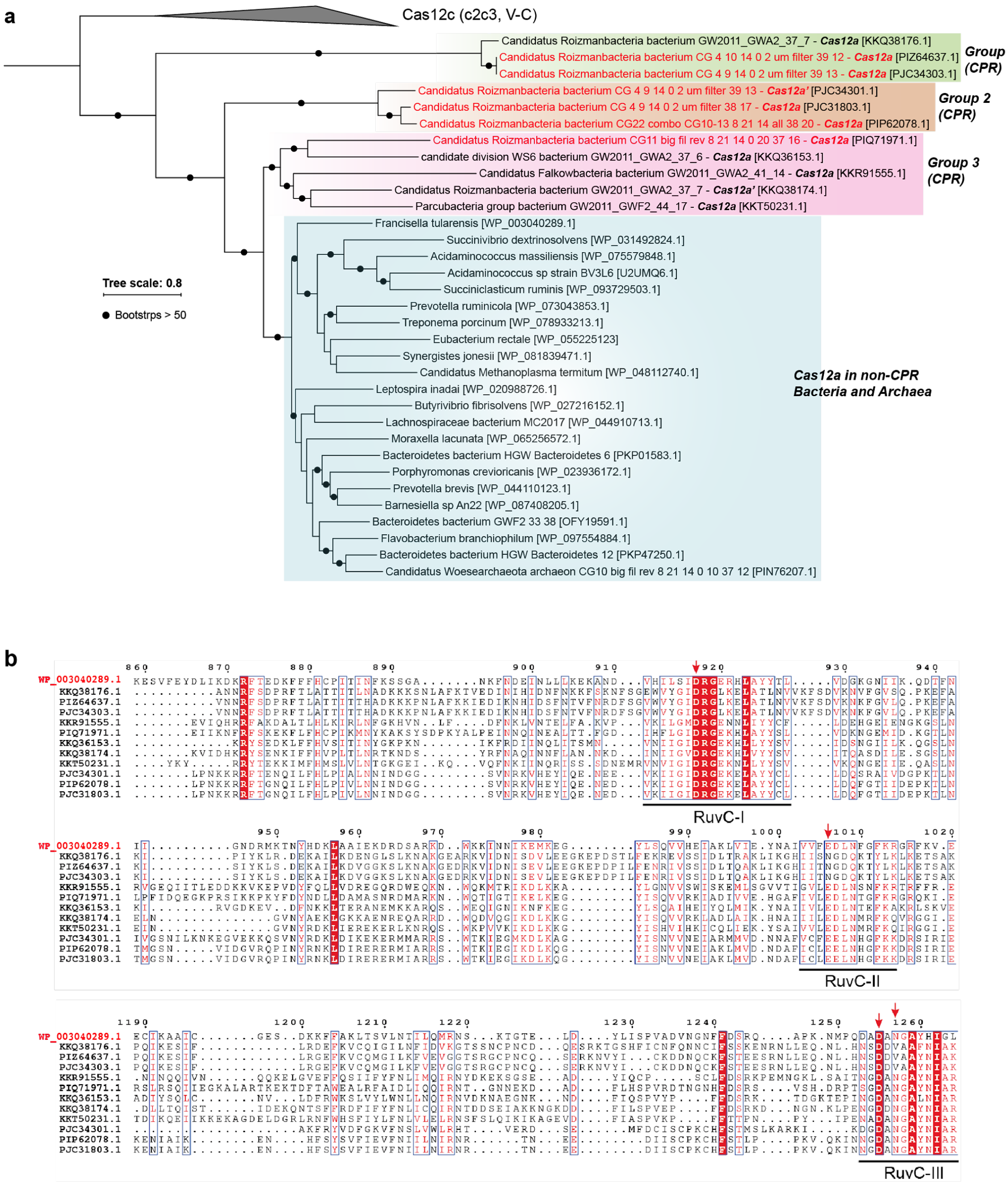

Figure S4 Cas12a proteins identified from published Roizmanbacteria genomes. (a) Phylogenetic analyses of Cas12a and Cas12a' proteins detected in CPR and non-CPR Bacteria and Archaea. The Cas12a proteins in Roizmanbacteria genomes (except the one identified by (Zetsche et al. 2015)) were predicted and annotated by NCBI search following detection of CRISPR-Cas system by CRISPRfinder. Published Cas12a proteins were retrieved (and manually confirmed) from NCBI by searching "cpf1" and/or "Cas12a", with each protein's corresponding Genbank ID shown in brackets. The Cas12a proteins in red are the ones reported in this study. (b) Analyses of RuvC domains indicating the substitution of catalytic residues (indicated by red arrows) in the RuvC-II and -III domains of Cas12a protein from genomes with both Cas12a and Cas12a'. The Cas12a protein in *Francisella tularensis* (WP_003040289.1) was used as reference for conserved domain alignment and analyses.

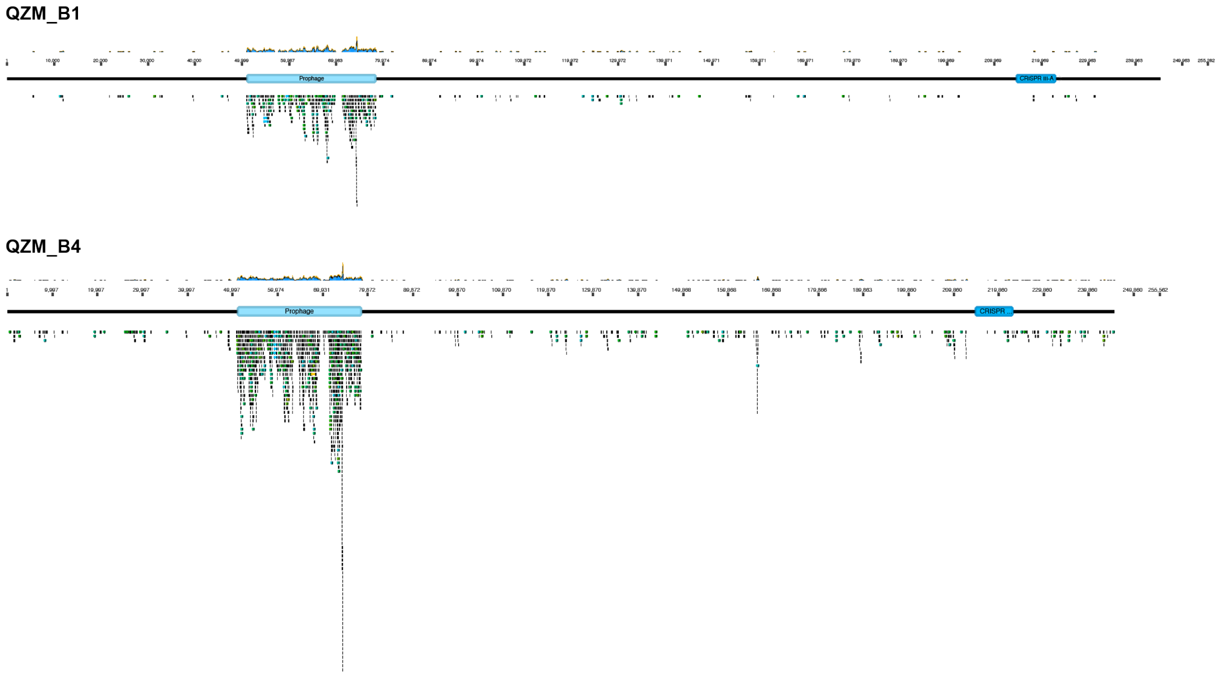

Figure S5 Read mapping profiles for the prophage region on aQZM_B3_Roizmanbacteria_33_70 scaffold. The reads from samples of QZM_B1 and QZM_B4 were mapped to the prophage region using bowtie2 with default parameters. Paired reads with two or more mismatches were removed using mapped.py (Brown et al. 2015). The mapping profiles were input into Geneious for visualization.

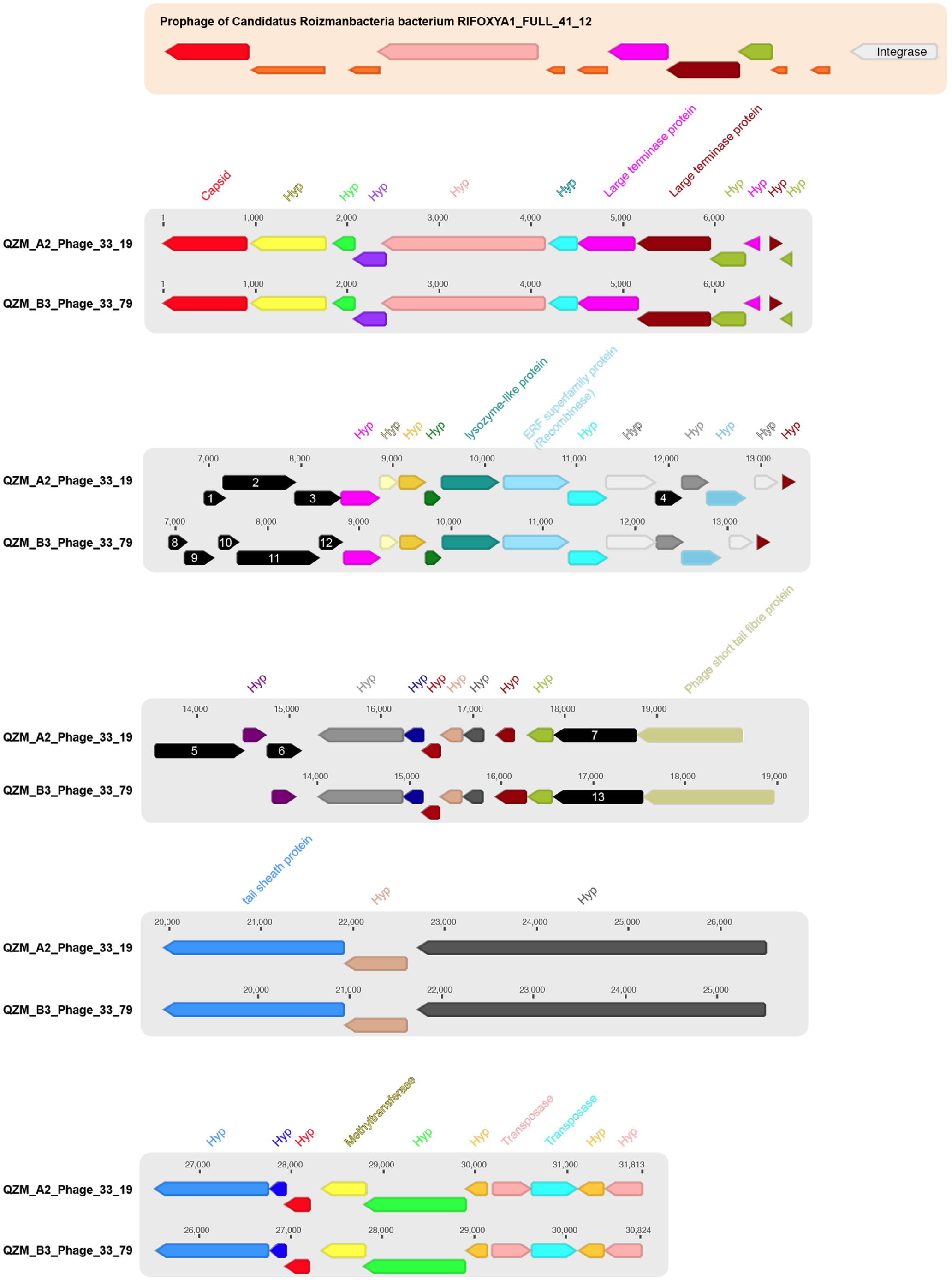

Figure S6 Comparative genomic analyses indicating the shared and non-shared ORFs between the two complete Roizmanbacteria-infecting phage genomes. The ORFs from the two phage genomes were compared against each other using BLASTp. ORFs with an alignment coverage > 80% and similarity > 80% were considered as homologues, and are indicated in the same color. The annotations of all shared ORFs are listed above the genes. Please see Figure 4 in the main text for detailed information regarding non-shared ORFs. The prophage region of a published Roizmanbacteria that shares similar ORFs with these two phage genomes is shown at the top of the figure, homologues are indicated in the same colors.

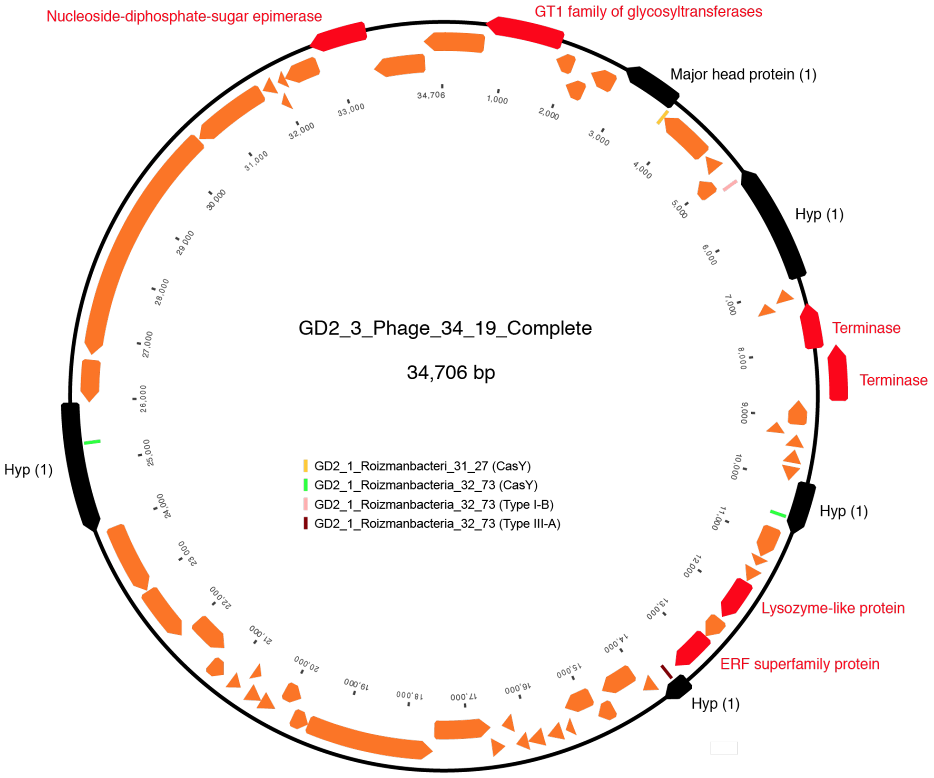

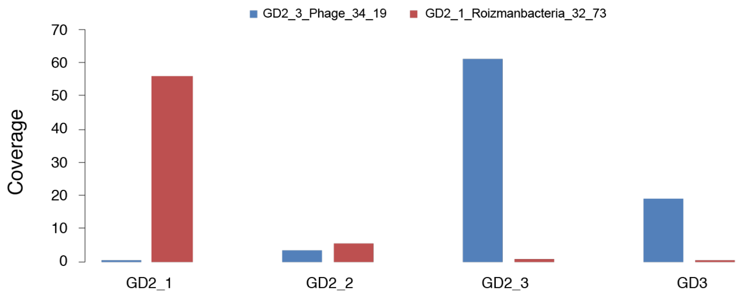

Figure S7 The complete phage genome reconstructed from GD samples. Predicted ORFs with annotations are shown in red. However, ORFs targeted by CRISPR spacers are shown in black. Genes without functional predictions are shown in orange. The targets of CRISPR-Cas spacers are indicated by colored rods; rod colors represent different systems. The coverages of GD_2_phage_34_19 and the organisms related to GD2_1_Roizmanbacteria_32_73 with fCasY in four GD samples are shown below.

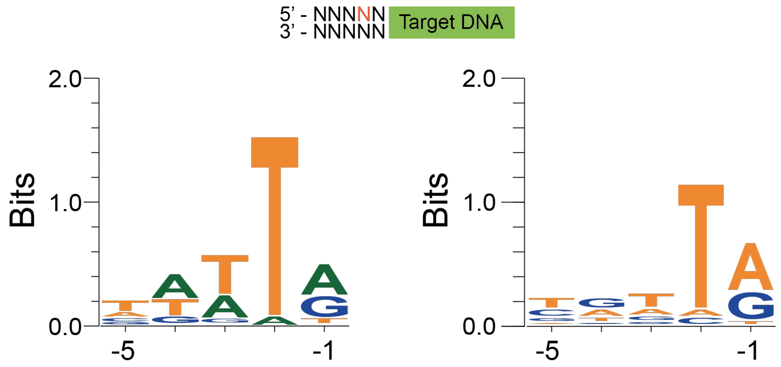

Figure S8 The PAM sequences of general CasY proteins (left) and the fCasY (right). This was performed with WebLogo using the upstream and downstream sequences of spacer targets as shown in Supplementary Table 6.

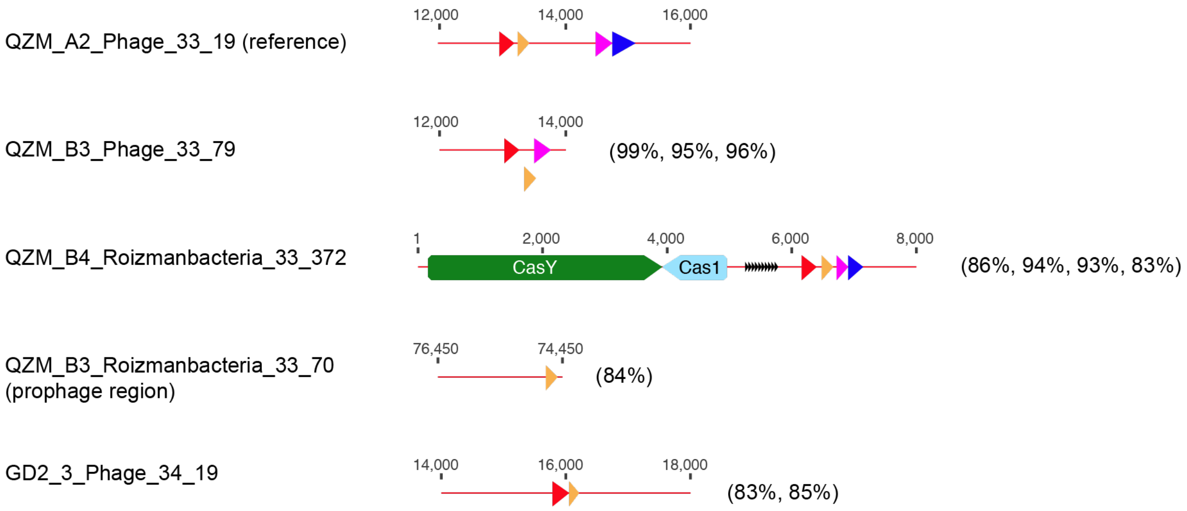

Figure S9 Homologues in QZM_B4_Roizmanbacteria_33_372 and Roizmanbacteria-infecting (pro)phage genomes reported in this study. The nucleotide sequence similarity values are indicated as %. Homologues share colors.
